# Erythrocyte Inosine Triphosphatase Activity Is Decreased in HIV-Seropositive Individuals

**DOI:** 10.1371/journal.pone.0030175

**Published:** 2012-01-17

**Authors:** Jörgen Bierau, Jaap A. Bakker, Jolanda A. Schippers, Janine A. C. Grashorn, Martijn Lindhout, Selwyn H. Lowe, Aimée D. C. Paulussen, Annelies Verbon

**Affiliations:** 1 Department of Clinical Genetics, Maastricht University Medical Centre, Maastricht, The Netherlands; 2 Department of Integrated Care, Maastricht University Medical Centre, Maastricht, The Netherlands; 3 Department of Medical Microbiology, Maastricht University Medical Centre, Maastricht, The Netherlands; 4 Department of Internal Medicine, Maastricht University Medical Centre, Maastricht, The Netherlands; 5 Department of Internal Medicine, Erasmus Medical Centre, Rotterdam, The Netherlands; South Texas Veterans Health Care System, United States of America

## Abstract

**Background:**

Inosine triphosphatase (ITPase) is encoded by the polymorphic gene *ITPA* and maintains low intracellular levels of the inosine nucleotides ITP and dITP. The most frequently reported polymorphisms are *ITPA* c.94C>A (rs 1127354) and *ITPA* c. 124+21 A>C (rs7270101). Some nucleoside-analogues used in the treatment of HIV-seropositive (HIV+) patients are potential substrates for ITPase. Therefore, the frequency of *ITPA* SNPs and ITPase activity were studied in a population of HIV+-patients.

**Methods:**

The study population consisted of 222 patients, predominantly Caucasian males, >95% using HAART. Erythrocyte ITPase activity was determined by measuring the formation of IMP from ITP. *ITPA* genotype was determined by sequencing genomic DNA. Distribution of ITPase activity, genotype-phenotype correlation and allele frequencies were compared to 198 control subjects. The effect of nucleoside analogues on ITPase activity was studied using lymphoblastic T-cell cultures and human recombinant ITPase. Enzyme kinetic experiments were performed on erythrocyte ITPase from HIV+ patients and controls.

**Results:**

No difference was observed in the allele frequencies between the HIV+-cohort (± HAART) and the control population. HIV+ carriers of the wild type and *ITPA* c.94C>A had significantly lower ITPase activities than control subjects with the same genotype (*p*<0.005). This was not observed in *ITPA* c. 124+21 A>C carriers. Nucleoside analogues did not affect ITPase activity in cell culture and human recombinant ITPase. Conclusion: *ITPA* population genetics were identical in HIV+ and control populations. However, the majority of HIV+-patients had decreased erythrocyte ITPase activity compared to controls, probably due to decreased amounts of ITPase protein. It seems unlikely that ITPase activity is decreased due to nucleoside analogues (HAART). Long-term effects of HIV-infection altering ITPase protein expression or stability may explain the phenomenon observed.

## Introduction

*ITPA* (OMIM 147520) encodes the enzyme inosine-5′-triphosphate pyrophosphohydrolase (inosine-5′-triphosphatase, ITPase), which is a housekeeping enzyme that controls the intracellular concentration of (deoxy)inosine-5′-monophosphate, (d)IMP, by catalysing the conversion of ITP and dITP to IMP and dIMP, respectively [1]. ITPase belongs to a family of nucleoside triphosphatase proteins that scavenge non-canonical nucleoside triphosphates that are potentially cyto- or genotoxic [2].

In humans, *ITPA* is a polymorphic gene of which multiple single nucleotide polymorphisms (SNPs) have been described. However, two SNPs, i.e. *ITPA* c.94 C>A (rs 1127354) and *ITPA* c. 124+21 A>C (rs 7270101) are found in all populations over the world with varying frequencies [3]. Both SNPs are associated with reduced ITPase activity [4], [5], the *ITPA* c.94 C>A substitution causes both an amino acid substitution (P32T) and causes missplicing whereas *ITPA* c. 124+21 A>C causes missplicing [6].

ITPase is expressed in all human tissues and has the highest mRNA expression in endocrine organs [7]. To this day, no overt causal clinical entity has been associated with deficiency of ITPase [8], [9], [10]. However, *ITPA* genotype may influence the clinical outcome of certain clinical conditions [11]. Until recently, pharmacogenetic consequences of *ITPA* have solely been implicated for the thiopurine drugs azathioprine and 6-mercaptopurine [4], [12]. Although the mechanism is still not fully elucidated, there is a tendency towards consensus that *ITPA* polymorphisms cause adverse drug reactions (ADR) in thiopurine therapy. More recently, *ITPA* polymorphisms have been associated with a protective effect against ribavirin induced anaemia in patients with hepatitis C [13]. Some nucleoside-analogues used in the treatment of HIV-seropositive (HIV+) patients are potential substrates for ITPase. Therefore, we performed a study in which the ITPase activity and *ITPA* genotype were determined in HIV+ patients. The ITPase activities were compared to established genotype-specific reference values. In addition, *in vitro* experiments were used to study effects of nucleoside analogue reverse transcriptase inhibitors on ITPase activity. The present paper focuses on an unexpected phenomenon we observed in our cohort of HIV-infected patients that raises deeper and more fundamental questions on the biological role of ITPase and the effect of HIV on human nucleotide metabolism.

## Results

### Patients and control population characteristics

The characteristics of the cohort of HIV+ patients are shown in table 1. Compared to the control population, the HIV+ patients were slightly younger than the control subjects and were predominantly male. The majority of patients were using HAART (212 out of 222) and had HIV RNA <40 copies/ml. A total of 15 patients had a sub-normal whole-blood haemoglobin concentration (<8.2 mmol Hb/l for men and <7.3 mmol Hb/l for women). The anaemic patients were 9 men with Hb 6.4–8.1 mol/l and 6 women with Hb 4.6–7.2. The HIV status of the control population is unknown. Assuming an incidence of HIV infection of 0.1% in the Dutch population [14], it cannot be excluded that one HIV+ individual was included in the control population. This would not change the results of our analysis.

**Table 1:** Patient and control subject characteristics.

	HIV+ patients	Reference populationITPase activity/genotype	Reference population genotyping	Genotype specific reference values
**N**	212	98	98	575
**Source**	MUMC HIV+ clinic	General hospital population (a)	Anonymous samples from healthy subjects (b)	Pharmacogenetic diagnostic services (c)
**Male/female**	185/27	49/49	49/49	-
**Median age (years)**	47	58		
**Predominant ethnicity**	Caucasian	Caucasian	Caucasian	Caucasian

Control populations consisted of (a) patients from the general hospital population, (b) healthy control subjects and (c) predominantly patients with inflammatory bowel or pulmonary disease.

### ITPA genotyping, population genetics and laboratory parameters

In order to determine the allele frequencies of the two prominent *ITPA* polymorphisms, the *ITPA* genotype was determined in all samples. The allele frequencies at positions *ITPA* c.94 and *ITPA* c.124+21 A>C in both populations were in Hardy-Weinberg equilibrium (table 2).The calculated allele frequencies were in accordance with previous reports for Caucasian populations [3], [5], [15]. Although at the *ITPA* c.94 locus there appeared to be a slightly higher incidence of adenosine residues in the HIV+ population, this was not statistically significant.

**Table 2:** *ITPA* allele frequencies in control and HIV+-populations.

Position	Reference sequence	Nucleotide	Controln = 182	HIVn = 203
***ITPA*** ** c.94**	**rs 1127354**	**C**	0.951	0.943
		**A**	0.049	0.057
***ITPA*** ** c.124+21**	**rs 7270101**	**A**	0.887	0.867
		**C**	0.113	0.133

Table 3 shows the *ITPA* genotypes identified in the HIV+-population. In accordance with what would be expected from the allele frequencies, most patients were carriers of the wild-type genotype, followed by *ITPA* c.121+21 A>C and *ITPA* c.94 C>A carriers, respectively. Only 7 patients had a genotype different from the mentioned genotypes.

**Table 3:** HIV patient parameters stratified by ITPA genotype.

*ITPA* genotype	All patients	Wild type	c.94C>A heterozygote	c.94C>A homozygote	c.124+21 A>C heterozygote	c.124+21 A>C homozygote	c.94C>A and c.124+21 A>C heterozygote
**N**	222	136	19	1	60	3	3
**Male/Female**	191/31	117/19	13/6	1/0	55/5	3/0	2/1
**Median age (years)**	47	47	45	41	46	48	42
**Range (years)**	21–79	21–79	24–62	-	27–65	47–63	36–75
**HIV RNA <40 copies/ml**	183	107	15	1	54	3	3
**HIV RNA >40 copies ml**	39	29	4	0	6	0	0
**Mean VL**	2595	11201	4322	-	6750	-	-
**Mean CD4+ lymphocytes ×10^6^/L**	574	583	541	416	723	389	863
**Using HAART**	212	128	18	1	59	3	3

No statistically significant relation between *ITPA* genotype and HIV RNA copy numbers, CD4+ lymphocytes count or use of antiretroviral therapy was observed (Table 3). Neither was a statistical significant correlation observed between ITPase activity, HIV RNA copy numbers, and CD4+ lymphocytes count (data not shown) No association between *ITPA* genotype, or ITPase activity with whole-blood haemoglobin content could be demonstrated. The haemoglobin concentration in the lysates prepared from the isolated erythrocytes was independent of whole-blood haemoglobin content and *ITPA* genotype.

### ITPA genotype and ITPase activity

No difference in ITPase activity could be shown for gender in the control population as well as in the HIV+ population (data not shown). The mean erythrocyte ITPase activity in the HIV+ population (3.55±1.69 mol/(mol Hb · h)) was significantly lower than in the control population (4.57±2.27 mol/(mol Hb · h)), *p*<0.001. Purine nucleoside phosphorylase (PNP) activity determined as reference enzyme activity, was within the reference range (71.3±6.7, 50–85 mol/(mol Hb · h)) in all HIV+ patients (78.6±11.1, 50–128 mol/(mol Hb · h)).

The ITPase activity of HIV+ individuals carrying either *ITPA* wild type or *ITPA* c.94 C>A was 20% lower than that of individuals with the same genotype from the control population (*p*<0.001). (table 4). The most pronounced decrease in ITPase activity was found in HIV+ heterozygous carriers of *ITPA* c.94 C>A. Instead of the expected 26% residual activity, the observed residual activity was 16% when compared to the HIV+ and control population wild type activities. Moreover, HIV+ carriers of *ITPA* c.94 C>A had 40% less ITPase activity than controls with the same genotype. Because in homozygous state the *ITPA* c.94 C>A polymorphisms leads to null activity in erythrocytes the effect could not be assessed for this genotype. The ITPase activity of both heterozygous and homozygous carriers of *ITPA* c.124+21 A>C did not differ significantly between both populations.

**Table 4:** ITPase activities stratified by genotype, compared between control and HIV+-population.

	Control		HIV+	
*ITPA* genotype	ITPase activity ± SD	% activity**	HIV+ ITPase activity ± SD	% activity**
**Wild type**	5.38±1.52	100	4.31±1.39*	80.1
**c.94 C>A heterozygote**	1.43±0.55	26.6	0.86±0.23*	16.0
**c.124+21 A>C heterozygote**	2.61±0.66	48.5	2.67±0.69	49.6
**c.94C>A homozygote**	0.00±0.05		0.03	
**c.124+21 C>C homozygote**	1.81±0.19	33.6	1.99±0.73	37.0

ITPase activity in mol IMP/(mol Hb × h).

*p<0.001.

**% activity wild type: residual ITPase activity relative to wild type ITPase activity.

### The effect of nucleoside analogues on ITPase activity

**Figure 1:**
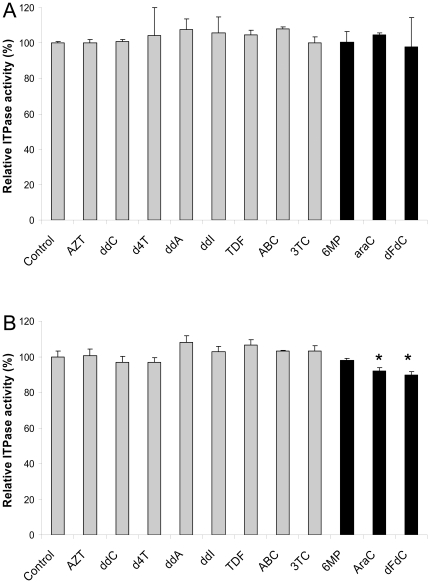
The effect of nucleoside analogues on ITPase activity. A: The effect of the addition of nucleoside analogues (10 µM) on the activity of hrITPase (2 µg). B: ITPase activity of MOLT-3 cells after 18 hr of incubation with 10 µM of the nucleoside analogues indicated; ziduvodine (AZT), zalcitabine (ddC), stavudine (d4T), dideoxyadenosine (ddA), didanosine (ddI), tenofovir (TDF), abacavir (ABC), lamivudine (3TC), 6-mercaptopurine (6MP), cytarabine (araC), gemcitabine (dFdC)* *p*<0.05.

Because the vast majority of the patients in the cohort were treated with HAART, one might argue that the nucleoside analogues in their circulation might have had an inhibitory effect on ITPase activity. The potential inhibitory effect of nucleotide or nucleobase analogues on ITPase was studied using human recombinant ITPase (figure 1A). After pre-incubation of 0.2 µg of hrITPase with 10 µM of either ziduvodine (AZT), zalcitabine (ddC), stavudine (d4T), dideoxyadenosine (ddA), didanosine (ddI), tenofovir (TDF), abacavir (ABC), lamivudine (3TC), 6-mercaptopurine (6MP), cytarabine (araC), or gemcitabine (dFdC) no significantly altered ITPase activity was observed.

The effect of exposure to nucleoside analogues on ITPase activity was assessed by incubating MOLT-3 lymphoblastic T-cells for 18 hrs with 0–100 µM of the panel of analogues. MOLT-3 cells had the wild type genotype for *ITPA*. The cytotoxic analogues araC and dFdC induced a significant decrease of ∼10% of ITPase activity. ITPase activity was not affected by the antiretroviral compounds and 6-MP (figure 1B, displaying the results of incubation with 10 µM analogue).

## Discussion

The nucleotide triphophosphates of nucleoside-analogue reverse-transcriptase inhibitors that are an integral part of HAART, are potential substrates for ITPase [1]. That prompted us to study the frequency of *ITPA* polymorphisms and the erythrocyte ITPase activity in a cohort of HIV-seropositive patients. We describe here the unexpected phenomenon that ITPase activity is reduced in the majority of patients infected with HIV.

The control population and HIV+ populations were not statistically different with respect to population genetics, and the allele frequencies were as previously reported for Caucasian populations [3], [5], [15]. This makes a hypothetical predisposition of carriers of *ITPA* polymorphisms to sustained HIV infection unlikely. Because the enzyme activity is a measure for the amount of functional ITPase protein present, it seems probable that the erythrocytes of HIV+ individuals contained less functional ITPase protein than those of controls. As of yet, it is unclear whether less ITPase protein was synthesised or that the stability of the protein was reduced. It is also possible that ITPase enzyme activity might be decreased in circulating red cells due to an aging erythrocyte population as a result of decreased bone marrow red cell production in HIV patients.

The decreased ITPase activity did not appear to be secondary to anaemia, as only <7% of patients were mildly anaemic and in these patients ITPase activity was independent of whole-blood haemoglobin concentration. Although *ITPA* SNPs appear to prevent ribavirin induced anaemia in patients with hepatitis C [13], in our HIV+-cohort anaemia was not associated with any of the *ITPA* SNPs. However, since our HIV+ cohort was relatively small it seems premature to draw a final conclusion about the relationship between *ITPA* polymorphisms and anaemia in HIV+ patients using antiretroviral therapy.

To determine whether or not use of HAART may lead to decreased ITPase activity, we explored the possibility that ITPase activity was decreased due to direct inhibition of the enzyme or by indirect effects of nucleoside analogues. We observed no direct effects of nucleoside analogue reverse transcriptase inhibitors either in cultured cells MOLT-3 cells exposed to these compounds or *in vitro* by addition of nucleoside analogues to the ITPase enzyme assay reaction mixture. This does not, however, exactly mimic the situation that occurs in patients receiving long-term treatment with HAART. Unfortunately, few HIV+ patients were not using antiretroviral therapy and firm conclusions cannot be drawn.

A shortage of nucleotide triphosphates, which may either be caused by mitochondrial dysfunction as seen in patients with HIV, or in theory by competition for phosphate esters between natural substrates and therapeutic nucleoside analogues, may cause a down-regulation of ITPase expression as a means of conserving triphosphate esters. This leaves unexplained why in case of hetero- or homozygosity of *ITPA* c.124+21 A>C the expression remained unaltered.

Interestingly, Jacobsson and colleagues described decreased level of thymidine kinase activity in lymphocytes of HIV+ patients and a generalised decrease in peripheral blood mononuclear cell thymidylate kinase was observed in HIV-infected individuals [16], [17]. Since only a fraction of all PBMC is infected with HIV, these authors concluded that this phenomenon was a generalised effect. In addition, HIV-infected T-lymphocytes were unable to biosynthesise ribonucleotides upon stimulation and consequently died [18]. Despite the fact that these studies were performed in white blood cells, these observations show intriguing parallels with our observation in eryhtrocytes, and suggest the possibility that there may exist a common mechanism by which HIV interferes in nucleotide metabolism.

With respect to the correlation between *ITPA* genotype and ITPase activity, the effect of the *ITPA* c.94 C>A polymorphism on ITPase activity is thought to be an accumulation of catastrophic events. *ITPA* is transcribed into three mRNA transcripts, of which the full length transcript is the major gene product for the wild type genotype. In the *ITPA* c.94 C>A carriers and homozygotes a transcript lacking exons 2 and 3 is prominent [6], resulting in less full length mRNA encoding the protein. Moreover, the mutation codes for an amino acid substitution, giving rise to a mutated protein having reduced to normal ITPase activity and, more relevant, decreased stability [19], [20]. It may be speculated that HIV itself or a factor secondary to HIV infection or treatment also affects expression of *ITPA*, activity or stability of the protein.

In conclusion, HIV-infected individuals carrying either *ITPA* wild type or c.94 C>A have reduced erythrocyte ITPase activity, most likely due to decreased amounts of active protein being present. This phenomenon was not observed in patients carrying *ITPA* 124+21 A>C. Long-term effects of HIV-infection altering protein expression or stability might explain the phenomenon observed.

## Materials and Methods

### Patients and control subjects

HIV-seropositive patients, visiting the HIV outpatient clinic of the Maastricht University Medical Centre (MUMC) aged 18 years and older were eligible for inclusion in the study. All consecutive 222 patients asked for participation consented. A reference population consisting of 98 anonymous samples from the general hospital population was used for determination of both ITPase activity and *ITPA* genotyping. Together, with another 98 anonymous DNA samples from healthy donors reference population genetics were determined. The two subpopulations proved to be statistically similar. Genotype specific erythrocyte ITPase reference values were determined using samples of which ITPase activity and *ITPA* genotype were known. This included the anonymous cohort and data obtained through our diagnostic pharmacogenetic services for patients with e.g. inflammatory bowel disease, or inflammatory pulmonary disease [21], [22]. All control samples were used according to the code for proper use of human tissue as formulated by the Dutch Federation of Medical Scientific Societies. The study was approved by the Medical Ethics Committee of the MUMC and all patients signed written informed consent.

### Materials

Full length recombinant human ITPase (hrITPase) from an E.coli expression system was purchased from Abnova (Bioconnect, Huissen, The Netherlands). Nucleoside analogues were obtained from Sigma (Zwijndrecht, The Netherlands). All other chemicals were of the highest grade. HPLC separation were performed using a Supelcosil LC-18S column (Sigma, Zwijndrecht, The Netherlands), with an Alliance Separation system (Waters, Etten-Leur, The Netherlands) equipped with a Jasco Multi-Wavelength detector (Jasco Benelux, Ijsselstein, The Netherlands). Data were analysed with Totalchrom data acquisition software (Perkin-Elmer, Groningen, The Netherlands). Haemoglobin was measured on a Coulter LH-750 haematology analyser (Beckman Coulter, Mijdrecht, The Netherlands).

### Cell culture and handling

MOLT-3 cells were obtained from the American Type Culture Collection (Manassas, VA). The cells were maintained in Dulbecco's Modified Eagles Medium, supplemented with 10% foetal calf serum, 2 mM L-Glutamine, 50 IU/ml penicillin and 0.2 mg/ml gentamycin at 37°C in humidified air with 5% CO_2_. Nucleoside analogues were added to the medium and after the appropriate incubation time the cells were harvested and washed by centrifugation.

### Enzyme activity assays

The ITPase activity in erythrocytes was determined as previously described [23]. Briefly, 25 µl of erythrocyte lysate, corresponding with approximately 45 nmol Hb, was incubated with 2.00 mM ITP, 50 mM MgCl_2_ in 100 mM Tris pH 7.4 in a final volume of 200 µl. The formation of IMP from ITP was quantified by using ion-pair HPLC method with UV detection. ITPase activity was expressed as moles of IMP formed from ITP in one hour per mol haemoglobin.

In assays with hrITPase, 0.2 µg of hrITPase was incubated with 10 µM of the appropriate nucleoside analogue under the same conditions as the routine ITPase assay.

The Purine nucleoside phosphorylase (PNP) activity was measured as a control assay for lysate quality. It was determined by incubating 15 µl of a ten-fold diluted erythrocyte lysate, corresponding with approximately 4.5 nmol Hb, with 7.5 mM inosine in 100 mM NaP_i_ buffer pH 6.6 for 30 minutes at 37°C. Further sample handling and activity measurement using chromatographic separation of the compounds of interest was as described above. PNP activity was expressed as moles of hypoxanthine formed from inosine in one hour per mol haemoglobin.

### DNA isolation and *ITPA* Genotyping

Genomic DNA was isolated from buffy coats using the Wizard Genomic DNA purification kit (Promega, Madison, WI). All DNA samples were genotyped for the polymorphisms *ITPA* c.94C>A (p.Pro32Thr, rs1127354) and c.124+21A>C (rs7270101). Wild type (wt) genotype was defined as no polymorphisms being detected at both positions. M13-tagged (underlined) primers forward 5′-TGTAAAACGACGGCCAGTCTTAGGAGATGGGCAGCAG and 5′-CAGGAAACAGCTATGACCCACAGAAAGTCAGGTCACAGG reverse were used in a PCR reaction of 10 µl. The resulting 241 bp PCR product was purified and directly sequenced in both directions using the Big Dye Terminator kit and analysed on an ABI 3720 Genetic Analyzer (Applied Biosystems, Carlsbad, CA). The sequence was aligned with *ITPA* reference sequence NM_033453.2 and genotypes were determined.

### Statistical analyses

Data are presented as mean values ± SD. ITPase activities in groups were compared using independent t-test and one-way Anova. Allele frequencies were compared using Chi-square test. Paired samples were analysed using Student's t-test. P-values<0.05 were considered to be significant.

## References

[1] Bierau J, Lindhout M, Bakker JA (2007). Pharmacogenetic significance of inosine triphosphatase.. Pharmacogenomics.

[2] Galperin MY, Moroz OV, Wilson KS, Murzin AG (2006). House cleaning, a part of good housekeeping.. Mol Microbiol.

[3] Marsh S, King CR, Ahluwalia R, McLeod HL (2004). Distribution of ITPA P32T alleles in multiple world populations.. J Hum Genet.

[4] Marinaki AM, Ansari A, Duley JA, Arenas M, Sumi S (2004). Adverse drug reactions to azathioprine therapy are associated with polymorphism in the gene encoding inosine triphosphate pyrophosphatase (ITPase).. Pharmacogenetics.

[5] Sumi S, Marinaki AM, Arenas M, Fairbanks L, Shobowale-Bakre M (2002). Genetic basis of inosine triphosphate pyrophosphohydrolase deficiency.. Hum Genet.

[6] Arenas M, Duley J, Sumi S, Sanderson J, Marinaki A (2007). The ITPA c.94C>A and g.IVS2+21A>C sequence variants contribute to missplicing of the ITPA gene.. Biochim Biophys Acta.

[7] Lin S, McLennan AG, Ying K, Wang Z, Gu S (2001). Cloning, expression, and characterization of a human inosine triphosphate pyrophosphatase encoded by the itpa gene.. J Biol Chem.

[8] Vanderheiden BS (1969). Genetic studies of human erythrocyte inosine triphosphatase.. Biochem Genet.

[9] Fraser JH, Meyers H, Henderson JF, Brox LW, McCoy EE (1975). Individual variation in inosine triphosphate accumulation in human erythrocytes.. Clin Biochem.

[10] Vanderheiden BS, Bora G (1980). Erythrocyte ITP pyrophosphohydrolase in chronic paranoid and undifferentiated schizophrenics: a biological difference.. Biochem Med.

[11] Bakker JA, Bierau J, Drent M (2010). A role for ITPA variants in the clinical course of pulmonary Langerhans' cell histiocytosis?. Eur Respir J.

[12] von Ahsen N, Armstrong VW, Behrens C, von Tirpitz C, Stallmach A (2005). Association of inosine triphosphatase 94C>A and thiopurine S-methyltransferase deficiency with adverse events and study drop-outs under azathioprine therapy in a prospective Crohn disease study.. Clin Chem.

[13] Fellay J, Thompson AJ, Ge D, Gumbs CE, Urban TJ (2010). ITPA gene variants protect against anaemia in patients treated for chronic hepatitis C.. Nature.

[14] Gras L, Van Sighem AI, Smit C, Zaheri S, Schuitemaker H (2009).

[15] Atanasova S, Shipkova M, Svinarov D, Mladenova A, Genova M (2007). Analysis of ITPA phenotype-genotype correlation in the Bulgarian population revealed a novel gene variant in exon 6.. Ther Drug Monit.

[16] Jacobsson B, Britton S, Tornevik Y, Eriksson S (1998). Decrease in thymidylate kinase activity in peripheral blood mononuclear cells from HIV-infected individuals.. Biochem Pharmacol.

[17] Jacobsson B, Britton S, He Q, Karlsson A, Eriksson S (1995). Decreased thymidine kinase levels in peripheral blood cells from HIV-seropositive individuals: implications for zidovudine metabolism.. AIDS Res Hum Retroviruses.

[18] Bofill M, Fairbanks LD, Ruckemann K, Lipman M, Simmonds HA (1995). T-lymphocytes from AIDS Patients Are Unable to Synthesize Ribonucleotides de Novo in Response to Mitogenic Stimulation.. J Biol Chem.

[19] Herting G, Barber K, Zappala MR, Cunningham RP, Burgis NE (2010). Quantitative in vitro and in vivo characterization of the human P32T mutant ITPase.. Biochim Biophys Acta.

[20] Stepchenkova EI, Tarakhovskaya ER, Spitler K, Frahm C, Menezes MR (2009). Functional study of the P32T ITPA variant associated with drug sensitivity in humans.. J Mol Biol.

[21] Bakker JA, Drent M, Bierau J (2007). Relevance of pharmacogenetic aspects of mercaptopurine metabolism in the treatment of interstitial lung disease.. Current Opinion in Pulmonology Medicine.

[22] Bakker JA, Lindhout M, Dorland L, Bierau J (2009). A comparative study of inosine triphosphatase activity in fresh erythrocytes and dried blood spots.. Clinica Chimica Acta.

[23] Bierau J, Bakker JA, Lindhout M, van Gennip AH (2006). Determination of ITPase activity in erythrocyte lysates obtained for determination of TPMT activity.. Nucleosides Nucleotides Nucleic Acids.

